# Nutri-Score: The Most Efficient Front-of-Pack Nutrition Label to Inform Portuguese Consumers on the Nutritional Quality of Foods and Help Them Identify Healthier Options in Purchasing Situations

**DOI:** 10.3390/nu13124335

**Published:** 2021-11-30

**Authors:** Francisco Goiana-da-Silva, David Cruz-e-Silva, Catarina Nobre-da-Costa, Alexandre Morais Nunes, Morgane Fialon, Manon Egnell, Pilar Galan, Chantal Julia, Zenobia Talati, Simone Pettigrew, Ara Darzi, Fernando Araújo, Serge Hercberg

**Affiliations:** 1 Centre for Health Policy, Institute of Global Health Innovation, Imperial College of London, London SW7 2BX, UK; franciscogoianasilva@gmail.com (F.G.-d.-S.); a.darzi@imperial.ac.uk (A.D.); 2 Center for Innovation, Technology and Policy Research, Universidade de Lisboa, 1049-004 Lisboa, Portugal; db.cruz.silva@gmail.com (D.C.-e.-S.); anunes@iscsp.ulisboa.pt (A.M.N.); 3 Associação Portuguesa de Jovens Farmacêuticos (APJF), 1169-075 Lisboa, Portugal; catarina.sdr.nobre@gmail.com; 4 Sorbonne Paris Nord University, Inserm U1153, Inrae U1125, Cnam, Nutritional Epidemiology Research Team (EREN), Epidemiology and Statistics Research Center, University of Paris (CRESS), 93000 Bobigny, France; m.egnell@eren.smbh.univ-paris13.fr (M.E.); p.galan@uren.smbh.univ-paris13.fr (P.G.); julia@eren.smbh.univ-paris13.fr (C.J.); s.hercberg@eren.smbh.univ-paris13.fr (S.H.); 5 Public Health Department, Avicenne Hospital, AP-HP, 93000 Bobigny, France; 6 School of Psychology, Curtin University, Kent St, Bentley, WA 6102, Australia; zenobia.talati@curtin.edu.au; 7 The George Institute for Global Health, Newtown, Sydney, NSW 2042, Australia; spettigrew@georgeinstitute.org.au; 8 Centro Hospitalar e Universitário de São João, 4200-319 Porto, Portugal; fernando.fmf66@gmail.com

**Keywords:** Front-of-Pack Nutrition Label, nutritional quality, diet, public health, Portuguese consumers

## Abstract

Several studies have identified Front-of-Pack Nutrition Labels (FoPLs) as a promising strategy to improve the nutritional quality of consumers’ food choices and encourage manufacturers to offer healthier products. This study aims to fill the evidence gap regarding the most effective FoPL among the Portuguese population. In total, 1059 Portuguese participants were recruited through a web panel provider and asked to declare their intended food choices and to rank three sets of products (pizza, cakes and breakfast cereals) according to their nutritional quality, first in the absence of any labelling, and then with a FoPL displayed on-pack (five FoPLs tested). Finally, participants were asked to answer nine statements related to perceptions of FoPLs. Results showed that participants improved their food choices, depending on the FoPL and the food category. All FoPLs led to a higher percentage of correct responses on the ranking task compared to the no label condition. The Nutri-Score was among the FoPLs producing the greatest improvement across all food categories compared to the reference intakes (OR = 6.45 [4.43–9.39], *p*-value < 0.0001) and facilitating the highest percentage to correctly rank products according to nutritional quality. This study suggests that, among the available options, Nutri-Score is the most efficient FoPL to inform Portuguese consumers of the nutritional quality of foods and help them identify healthier options in mock purchasing situations.

## 1. Introduction

According to the Global Burden of Disease study, unhealthy diets are the leading risk factor contributing to the loss of healthy life years among the Portuguese population [1,2]. Overweight and obesity are present in over 50% of the Portuguese population [3]. Chronic diseases, which are associated with avoidable behavioural risk factors, and for which dietary habits are an important determinant [2], represent 86% of the total burden of disease [2,4]. Thus, there is a need for a new philosophy towards public health with increased investment in health promotion and a multi-stakeholder approach involving modifications of the food environment.

Several studies have shown that consumers, in general, are not able to fully understand and interpret food labels [5,6]. Data available for the Portuguese population support these results. A study, which was developed with the World Health Organization (WHO, Geneva, Switzerland), showed that 40% of Portuguese participants do not understand back of pack nutritional information [6]. Furthermore, among people with lower socioeconomic status, this figure increased to 60%. The study also found that FoPLs using colours led to better label comprehension.

FoPLs were first introduced in the 1980s/1990s with some being endorsed by countries and implemented as part of national nutrition prevention programs [7,8] (namely, the Green Keyhole has been used in the Nordic countries since the 1980s, the Multiple Traffic Lights (MTL) in the United Kingdom since 2004, the Warning symbol in Chile since 2016, the Health Star Rating system in New Zealand and Australia since 2014, and the Nutri-Score in France in 2017 and then by several other European countries). Furthermore, the Guideline Daily Amount, which became the Reference Intakes (RIs), was implemented by many manufacturers in 2006 as part of a voluntary initiative by agro-food industries.

Several studies identified FoPLs as a promising strategy to improve the nutritional quality of consumers’ food choices and encourage manufacturers to offer healthier products and improve the nutritional profile of existing products [9,10,11,12,13]. The WHO recommended the implementation of FoPLs as a highly cost-effective intervention, also known as ‘best-buy’ intervention, to prevent non-communicable diseases (NCD) [14] given their effectiveness as a nudging strategy [15].

Nonetheless, the existence of multiple FoPLs may generate confusion among consumers [16]. According to European Regulation, FoPLs are voluntary and different models may coexist within the same market [17]. However, discussions by the European Commission have been taking place since 2018 in order to modify the existing regulation towards a harmonised regulatory context [4]. International government interest in this area has been particularly felt through the inclusion of political discussion with regards to FoPLs within a Codex Alimentarius e-working group [18]. Finally, the European Commission decided to implement, as part of its Farm to Fork strategy in May 2020, a single harmonised nutrition FoPL for Europe in 2022.

Evidence suggests that FoPLs that use colours are the most efficient in providing nutritional information, especially among vulnerable populations [19,20]. These types of labels (such as the Multiple Traffic Lights in England or the Nutri-Score in France) [21,22,23,24] allow consumers to better understand the nutritional quality of foods and make healthier food choices.

In Portugal, several proposals of FoPLs have been brought forward to the Parliament. However, to date, none have received political approval. Various kinds of FoPLs can be found in Portuguese supermarkets: the Multiple Traffic Lights (MTL) initially developed by the Food Standards Agency in the United Kingdom, was adopted by the Portuguese retail chain Continente for its brand in 2009; the Reference Intakes format supported by FIPA (Federação das Indústrias Portuguesas Agro-Alimentares) is also visible on many products (e.g., Pingo Doce retail brand). The Ipsos European Union study showed that Portugal was one of six European countries with the most food labelling schemes [25]. Still, as a result of the multiplicity of proposals brought forward from different political parties, the Portuguese Parliament recommended that the Portuguese Government assessed a simplified FoPL model as an alternative to the Traffic Light System.

This article aims to help fill the evidence gap regarding the most effective FoP Labelling system among the Portuguese population by assessing consumer responses to different FoPLs in a Portuguese sample. This study is part of the second wave of the international comparative experimental study initially conducted across 12 countries and then on six additional European countries that evaluated consumers’ responses to five different FoPLs in the form of perceptions, objective understanding and food choices (FOP-ICE study [26,27]). 

## 2. Materials and Methods

### 2.1. Population Sample

A total of 1059 participants were recruited through an ISO accredited international web panel provider (Pureprofile) using advertising and word-of-mouth referrals. Quotas for age (a third in each category: 18–30 years, 31–50 years, over 51 years), sex (50% women) and household income level (a third in each category: low, medium and high) were applied. Income levels were calculated by estimating the median annual household income in Portugal (from national statistical databases) and creating a bracket of 33% around this figure. This represented the “medium” income band. Anything below or above this figure was considered as low- or high-income, respectively. Participants gave their electronic consent and, during an online questionnaire, were asked to provide socio-demographic, lifestyle and nutrition-related information, such as sex, age, monthly household income, educational level, involvement in grocery shopping, self-estimated diet quality, and self-estimated level of knowledge in nutrition. Individuals were excluded if they reported never or rarely buying at least two of the three food categories studied (pizzas, cakes and breakfast cereals)—response options were “always”, “often”, “sometimes” and “never”.

Data collection took place in the second wave of the FOP-ICE study [27] and received approval from the Curtin University Human Research Ethics Committee (approval reference: HRE2017-0760) and the Institutional Review Board of the French Institute for Health and Medical Research (IRB Inserm n_17-404 and 17-404 bis).

### 2.2. Stimuli

**Figure 1:**
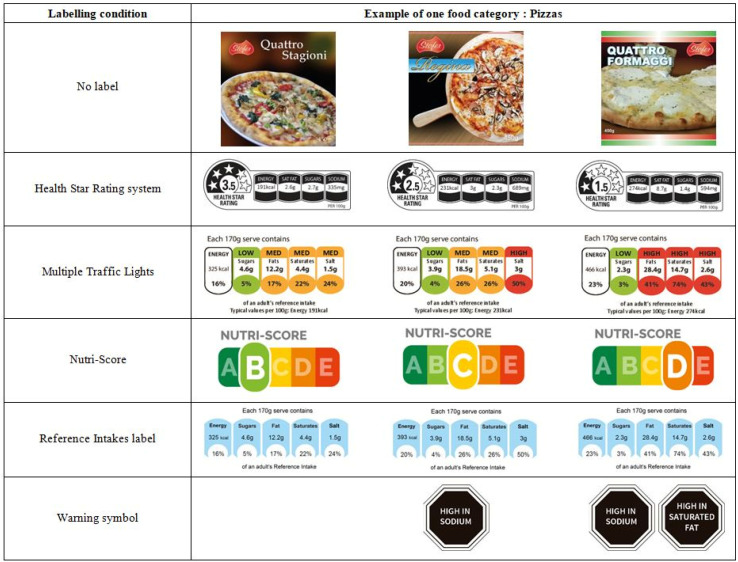
Example of the set of three pizzas with the corresponding labelling conditions.

Participants were presented with virtual fictitious food products (designed specifically for this study) (Figure 1). These products (pizzas, cakes and breakfast cereals) were selected due to two main reasons. Firstly, these products are consumed in all the countries where the FOP-ICE study was conducted (including Portugal). Secondly, there was high variability in nutritional quality within the food category. 

The mock products, including the brand, were specifically created to resemble real food products. However, the product creation and design accounted for factors that could interfere with respondents’ judgment, such as brand and product loyalty and/or familiarity, as well as purchase habits. A zoom function was available for participants to enlarge any area of the image of the mock package.

Within each category, three products were presented to each participant. Each one of these products had a distinct nutritional quality profile (lower, intermediate and higher). Only the FoPL was provided and there were no other labels (e.g., organic label, gluten-free). 

### 2.3. Procedure

Firstly, participants were confronted with the sets of three products without any label (i.e., No Label Condition). For each of the three food categories, participants were asked to undergo a food choice task and a ranking task. Secondly, all participants were randomised to one of the five FoPLs groups (Health Star Rating (HSR), Multiple Traffic Lights (MTL), Nutri-Score, Reference Intakes (RIs) or Warning symbol) (Figure 1), with a balanced repartition between the five groups (i.e., approximately 200 participants per group). The two previous tasks were then repeated with the randomly assigned label (FoPL condition). Finally, participants were asked to answer nine statements related to several aspects of perception.

#### 2.3.1. No Label Condition

Participants were asked to choose which product they would buy among the three products of each set. The option “I wouldn’t buy any of these products” was also available. Participants were then asked to rank the products according to their nutritional quality. For each product, participants could select “1—Highest nutritional quality”, “2—Medium nutritional quality” or “3—Lowest nutritional quality”. The option “I don’t know” was also available. The two tasks were performed sequentially.

#### 2.3.2. FoPL Condition

Each participant was then presented with one of the five FoPLs, assigned by randomisation. They were asked to repeat the same tasks, but this time with a label on all the products. Participants were not aware that they would be seeing the products twice, or that a FoPL would be present on the second viewing. The order of the products and categories was randomised between participants to avoid undesired order effects. The underlying nutritional ranking of the products was the same regardless of the type of label used. Participants were also asked if they recalled the FoPL to which they were exposed. 

#### 2.3.3. Perception

Participants were asked to answer nine statements on a 9-point Likert scale from 1 (strongly disagree) to 9 (strongly agree). These statements were related to perceptions of FoPLs; in particular ease of understanding (e.g., “this label is easy to understand”, “this label is too long to understand”), label visibility (“This label does not stand out”), appreciation (“I like this label”) and trust (“I trust this label”).

### 2.4. Statistical Analysis

#### 2.4.1. Food Choice and Objective Understanding

For food choice, a score between 1 and 3 points was assigned to each product. A score of 3 for the product with the highest nutritional quality, 2 for the intermediate product, 1 for the product with the lowest nutritional quality. Participants’ objective understanding, corresponding to the ability of consumers to understand the information provided by labels in the way expected by its designers, was evaluated by calculating the difference between the ranking tasks in the no label and FoPL conditions [28]. For the objective understanding analyses, for each food category, participants were allocated 1 point if they ranked all the products in the expected order according to the nutritional quality of products, 0 if the answer was “I don’t know” and −1 point if they ranked at least one product out of order.

For each food category, a score was calculated by subtracting the choice/ranking score in the no label condition from the score in the FoPL condition. For each food category and for each task, a discrete score between −2 (which was considered as the highest deterioration) and +2 points (the highest improvement of the food choice) was calculated. Participants’ scores were summed across the three categories, resulting in a final global discrete score ranging from −6 to +6. Only responses from participants who selected a product in both the no label and FoPL conditions were included.

Sensitivity analyses for objective understanding were performed by excluding participants who answered “I don’t know” to the ranking task, drawing upon previously published methodology [27]. The responses of participants who declared never purchasing products from one of the three food categories were excluded from the corresponding food category. 

Ordinal logistic regression models were used to evaluate the association of FoPL with better food choices and with the ability to correctly rank products. Since previous literature shows that the RIs are the least efficient FoPL [20], this label was used as the reference category. Covariates considered for the regression models were sex, age, educational level, household income, involvement in grocery shopping, responses to the question “Do you remember having seen this label during the survey?” and self-estimated nutritional knowledge and diet quality. The analyses were done by food category and for all categories. Interactions between FoPLs and these covariates were also tested (a *p*-value below 0.05 was considered statistically significant).

#### 2.4.2. Perception

For perception analyses, participants who gave the same score to all perception questions were excluded. An exception was made for those who gave a score of 5, a neutral perception, on all items. For each perception question, the mean and standard deviation was computed per FoPL. Furthermore, a Principal Component Analysis (PCA) was conducted to investigate the contribution of the different questions to the overall label perception. For the PCA, “This label is confusing”, “I like this label”, “This label does not stand out”, “This label is easy to understand”, “This label takes too long to understand”, “This label provides me the information I need” and “I trust this label” were considered as active variables. The FoPL itself was considered as a qualitative supplementary variable. The contribution and coordinates of each variable on each dimension were computed and the label was mapped on the axes as an illustrative variable. The analysis of a test value allows testing the significance of the deviation from the origin of the qualitative variable. This difference can be considered significant at the 95% level if the test value is greater than or equal to 2 in absolute value [29]. 

A *p*-value below or equal to 0.05 was considered statistically significant. Statistical analyses were carried out for all food categories combined and for each individual food category, using SAS Software (version 9.3, SAS Institute Inc., Cary, NC, USA). Analysis on perception was carried out using R software (version 3.4.4, R Foundation, Vienna, Austria).

## 3. Results

The sample included 50% of women, 34% of participants between 18 and 30 years old, 33% with a low level of income, as per quota sampling, 34% with a primary or secondary educational level, 40% with an undergraduate degree, and 60% responsible for grocery shopping. A total of 14% declared having a very or mostly unhealthy diet, and 10% were not very knowledgeable about nutrition or did not know anything, whilst 64% felt they were somewhat familiar with nutrition. A total of 62% declared having seen the FoPL during the survey. The Warning symbol and the HSR were the two FoPLs with the lowest recall rate (47% and 52% respectively).

### 3.1. Food Choice and Objective Understanding

**Figure 2:**
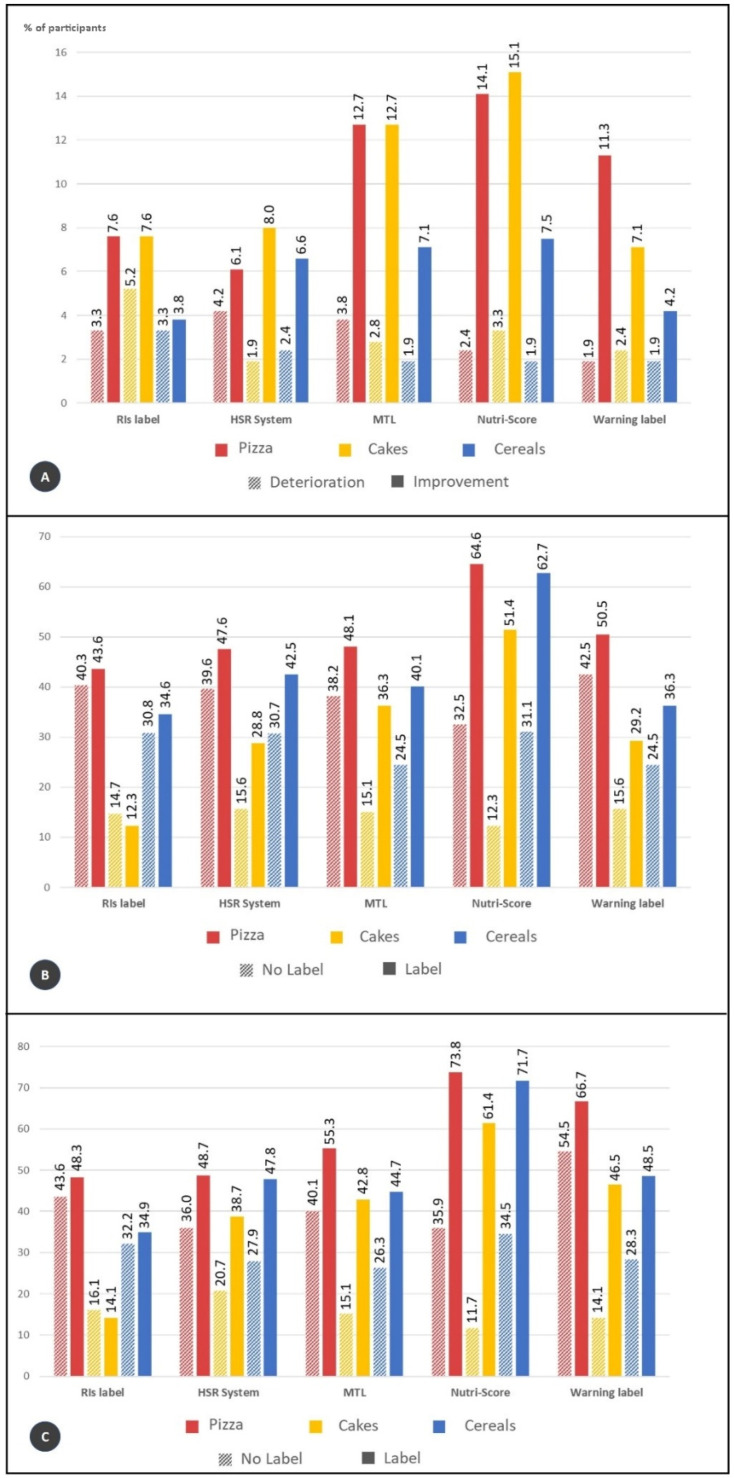
(**A**) Percentages of participants whose food choices deteriorated and improved; (**B**) Percentages of participants providing correct answers for ranking tasks; (**C**) Percentages of participants providing correct answers for ranking task only among participants who recalled seeing the label during the survey.

Between 3.8% and 15% of participants improved their food choices, depending on the label and food category. Improvement was always higher than deterioration, per FoPL and per food category, which varied between 1.9% and 5.2% depending on the label and the food category. Nutri-Score and the MTL were the FoPLs with the higher percentages of improvement across all food categories (Figure 2A).

For objective understanding, the change in the number of correct responses across the three food categories from the no label to the FoPL condition was computed for each participant. For the three food categories, the Nutri-Score was the FoPL with the highest increase of the number of correct answers compared to no label with a relative increase ranging from 99% for pizzas to 319% for cakes and 102% for breakfast cereals. 

The MTL was the FoPL demonstrating the second-best performance for the three categories, but with a much lower effect compared to the Nutri-Score. The relative increase of correct answers compared to the no label condition ranged from 26% for pizzas to 141% for cakes and 63% for breakfast cereals. For the other FoPLs, results were contrasted depending on the food category; however, RIs showed the lowest performance in each category.

Regarding the ranking task, all FoPLs led to a higher percentage of correct responses compared to the no-label condition. Nutri-Score was the FoPL with the highest improvement on the ability to correctly rank products according to nutritional quality, reaching between 32% and 39% of improvement (Figure 2B).

Analyses were also conducted among participants recalling having seen the FoPL only during the survey. The Nutri-Score labelling showed higher results for the three food categories but this time followed by the Warning symbol (Figure 2C).

MTL, Nutri-Score and Warning symbols were more efficient than RIs in helping consumers improve their food choices. Nutri-score was the most efficient FoPL in helping consumers improve the nutritional quality of food choices (OR = 1.98 [1.26–3.12], *p*-value = 0.003) (Table 1).

No quantitative interaction between labels and individual characteristics was found (data not shown).

According to the association between FoPL and the ability to correctly rank food products matching their nutritional quality, the Nutri-Score was the FoPL associated with the highest improvement in participants’ ability to correctly rank products (OR = 6.45 [4.43–9.39], *p*-value < 0.0001) compared to the RIs, followed by the MTL, the HSR and then the Warning symbol. For the three food categories, the Nutri-Score showed stronger performances, while the other labels varied in performance depending on the food category (Table 2).

As for the sensitivity analyses, results show that excluding participants selecting “I don’t know” led to similar findings for the FoPLs’ performance regarding objective understanding, with even larger effect magnitudes. However, the Warning symbol was the second label to perform best after the Nutri-Score among participants recalling having seen the label during the survey (Table 3).

No interaction between FoPLs and individual characteristics was found.

### 3.2. Perception

The results regarding the comparison of the average score and the standard deviation of each statement by FoPL were globally homogenous, with less than 1 point of score between different FoPLs for each statement, except for the statement “This label does not stand out” where the average score for the Nutri-Score was equal to 3.7 and for the Warning symbol equal to 5.2 (Table 4).

**Figure 3:**
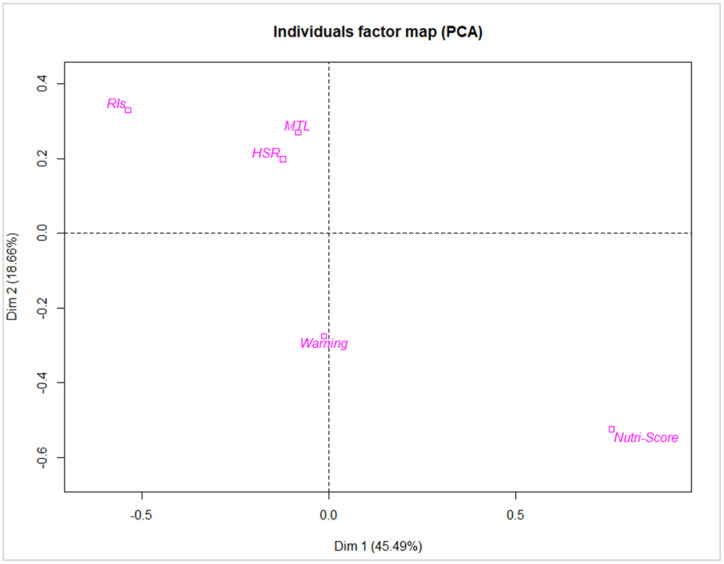
Principal Component Analysis map.

Two dimensions were retained, explaining respectively 46% and 19% of the variance. After evaluating contributions of active variables, the first dimension (horizontal axis) represented rather preference (including the statements “This label is confusing”, “This label is easy to understand” and “This label takes too long to understand”) whereas the second dimension represented trustworthiness (including the statements “This label provides me the information I need”, “I trust this label”) (Figure 3).

After analysing the PCA and v-test, Nutri-Score and the RIs were opposed on the two dimensions. According to the first dimension, Nutri-Score was less confusing and faster to understand compared to the RIs. According to the second dimension, the RIs provided more information and were more trusted by consumers compared to the Nutri-Score.

## 4. Discussion

While evidence shows that Portuguese consumers use labelling information to make their purchasing decisions [30], roughly 40% of surveyed Portuguese consumers do not understand the back of pack labelling information [6]. Our study shows that FoPLs can increase the nutritional quality of food choices made by Portuguese consumers, which is in line with previous research [31]. In terms of objective understanding, we found that all FoPLs seemed to increase consumers’ understanding, however, the Nutri-Score improved consumers’ capacity to understand the nutritional quality of products the most. In terms of the quality of food choices, the Nutri-Score showed the greatest increase, therefore, has the most potential to help Portuguese consumers improve their diets. All FoPLs were well perceived by participants, however, the RIs were seen as more confusing and took too long to understand compared to the Nutri-Score.

The results of this study suggest that among the available options, the Nutri-Score, with its summary, graded, color-coded format, appears as the most efficient scheme to inform Portuguese consumers of the nutritional quality of foods and help them identify healthier options in purchasing situations. 

The data shown in this study are of particular relevance when taking into account the context in Portugal. The retail market leader in Portugal has been implementing an adapted MTL model for roughly ten years. This has led to the Portuguese population being well exposed to an MTL-like model [32]. In fact, within the sample of Portuguese consumers, Nutri-Score and the MTL were the FoPLs with the higher percentages of improvement across all food categories. Furthermore, when assessing the full sample, the Nutri-Score showed the largest improvement in the ability to correctly rank products according to nutritional quality, closely followed by MTL. 

The results of this study were somewhat expected due to existing data on the efficacy of Nutri-Score in France [33,34,35,36], in other Mediterranean countries like Spain [37], or Italy [38], as well as in many other European countries [20,26]. The similarities between the Portuguese, Spanish, Italian and French populations, their food environments and behaviours [39] may also explain these findings. The results of this study are consistent with previous findings, particularly with regard to objective understanding and food choices [20,27]. A study, which took place in over 20 stores, showed that summary models (such as Nutri-Score) are the most effective in improving consumer decision-making [36]. Furthermore, several experimental studies have provided similar findings [34,35].

An increasingly robust body of evidence suggests that the Nutri-Score significantly improves consumers’ ability to understand nutritional information and make healthier choices. Several food manufacturers and retailers have followed suit and deployed (or committed to deploying) the Nutri-Score on their products. In Portugal, some food industry players have also publicly announced the use of this FoPL. Furthermore, other Ministries of Health in Europe (i.e., France, Belgium, Spain, Switzerland, Germany, Luxembourg and the Netherlands) have endorsed the label and/or published national recommendations for its adoption. The Nutri-Score also has the support of numerous European consumer associations. 

In Portugal, unlike other European countries, little progress has been made in this area. Policy and regulatory levers lying outside the health sector, limited collaboration between different governmental areas, and vested interests within and outside of the health sector have deeply hampered any decisive action from being taken. This study, by using a randomization design to compare the effectiveness of five FoPLs, while combining three dimensions being the potential effect of the FoPLs on food choices, the understanding of labels and their perceptions by consumers brings new insights for public health policymakers in Portugal. Regarding existing evidence on FoPLs in Portugal, a Portuguese study conducting a health impact assessment of FoPLs as a public health measure did not conclude in any specific FoPL format recommendation [40]. Recent research found that MTL was the most preferred FoPL in a sample of Portuguese adults exposed to the same set of FoPLs as this study except for the Warning symbol [32]. However, this result may be explained by the familiarity of Portuguese consumers with MTL as discussed by the authors. Also, this Portuguese study highlighted that Nutri-Score was more adapted for all socioeconomic status subgroups compared to other FoPLs, consistent with findings from Egnell et al. [20] and is of particular interest considering that, as mentioned before, among people with lower socioeconomic status, 60% do not understand back of pack nutritional information [6]. 

Until now, a lack of evidence on the real impact of the use of foreign FoPL among the Portuguese population has been described as an obstacle to the Portuguese Government endorsing a single FoPL. The multiplicity of FoPLs on Portuguese food products may complicate their understanding and discourage their use by consumers [16]. Results of this study effectively fill the gap previously identified and reinforce the need for standardization of the FoPL used at a national level in order to put a stop to the confusion generated within consumers’ minds when exposed to several different FoPLs.

Some limitations of the study should be acknowledged. First, participants were recruited using quotas that are not specific to the Portuguese population which requires caution regarding the extrapolation of the results. Nevertheless, this allowed the inclusion of Portuguese participants from various sociodemographic profiles for whom FoPL effectiveness can vary, as well as comparisons of FoPL effectiveness between multiple countries within the FOP-ICE study. Second, as the meaning of the five FoPLs were not explained to participants, it may have led to an underestimation of the labels’ effects, especially for unknown FoPLs compared to the RIs or the MTL labels that can be found in Portuguese supermarkets. Additionally, participants were exposed to virtual food products where factors such as prices were not included. Future studies in real-life environments are needed to take into account Portuguese specificities in the case of FoPLs. Finally, the results could have been impacted by purchasing habits for the food categories used in the study but this bias was minimised by the use of fictional brands and the exclusion of the answers of participants who declared having never purchased one of the food categories.

## 5. Conclusions

Our study suggests that, among the available options, the Nutri-Score appears as the most efficient Front-of-Pack Nutrition Label to inform Portuguese consumers of the nutritional quality of foods and help them identify healthier options in purchasing situations. These findings are in line with the results of previous investigations performed on FoPLs and strengthen the body of evidence needed to harmonize FoPLs in Portugal for policymakers. Future studies could evaluate the effectiveness of these FoPLs on Portuguese consumers under real-life conditions. 

## Figures and Tables

**Table 1:** Association between FoPLs and change in the nutritional quality of food choices.

Food Category	*n*	HSR		MTL		Nutri-Score		Warning Symbol	
		OR (95% CI)	*p*	OR (95% CI)	*p*	OR (95% CI)	*p*	OR (95% CI)	*p*
All categories	1007	1.46 [0.92–2.32]	0.1	1.95 [1.24–3.07]	**0.004**	1.98 [1.26–3.12]	**0.003**	1.94 [1.22–3.08]	**0.005**
Pizza	864	1.00 [0.54–1.86]	1	1.56 [0.87–2.81]	0.1	1.92 [1.07–3.43]	**0.03**	1.99 [1.1–3.6]	**0.02**
Cakes	759	1.64 [0.87–3.06]	0.1	1.89 [1.02–3.49]	**0.04**	2.2 [1.2–4.03]	**0.01**	1.41 [0.75–2.68]	0.3
Breakfast Cereals	890	1.81 [0.87–3.77]	0.1	1.82 [0.87–3.8]	0.1	2 [0.96–4.13]	0.06	1.55 [0.72–3.34]	0.3

The reference for the multivariate ordinal logistic regression for the categorical variable ‘label’ was Reference Intakes. The multivariate model was adjusted on sex, age, educational level, level of household income, responsibility for grocery shopping, self-estimated diet quality, self-estimated nutrition knowledge level, and “Did you see this label during the online survey?”. HSR: Health Star Rating system; MTL: Multiple Traffic Lights; OR: Odds Ratio; CI: Confidence Interval. Bold values correspond to significant results (*p*-value ≤ 0.05).

**Table 2:** Association between FoPLs and the ability to correctly rank products according to nutritional quality.

Food Category	*n*	HSR		MTL		Nutri-Score		Warning Symbol	
		OR (95% CI)	*p*	OR (95% CI)	*p*	OR (95% CI)	*p*	OR (95% CI)	*p*
All categories	1059	2.02 [1.39–2.94]	**0.0002**	2.11 [1.46–3.05]	**<0.0001**	6.45 [4.43–9.39]	**<0.0001**	2.00 [1.37–2.91]	**0.0003**
Pizza	1031	1.42 [0.91–2.22]	0.1222	1.30 [0.84–2.01]	0.2470	3.67 [2.39–5.64]	**<0.0001**	1.33 [0.85–2.08]	0.2073
Cakes	1038	2.56 [1.65–3.97]	**<0.0001**	3.19 [2.07–4.93]	**<0.0001**	7.14 [4.61–11.07]	**<0.0001**	2.88 [1.85–4.48]	**<0.0001**
Breakfast Cereals	1027	1.58 [0.98–2.56]	0.0614	1.77 [1.11–2.83]	**0.0170**	3.68 [2.33–5.8]	**<0.0001**	1.56 [0.96–2.53]	0.0727

The reference of the multivariate ordinal logistic regression for the categorical variable ‘label’ was the Reference Intakes. The multivariate model was adjusted on sex, age, educational level, level of household income, responsibility for grocery shopping, self-estimated diet quality, self-estimated nutrition knowledge level, and “Did you see this label during the online survey?”. HSR: Health Star Rating system; MTL: Multiple Traffic Lights; OR: Odds Ratio; CI: Confidence Interval. Bold values correspond to significant results (*p*-value ≤ 0.05).

**Table 3:** Associations between FoPL exposure and ability to rank products among participants recalling seeing the label.

Food Category	*n*	HSR		MTL		Nutri-Score		Warning Symbol	
		OR (95% CI)	*p*	OR (95% CI)	*p*	OR (95% CI)	*p*	OR (95% CI)	*p*
All categories	656	2.17 [1.37–3.42]	**0.001**	2.70 [1.76–4.15]	**<0.0001**	8.11 [5.18–12.7]	**<0.0001**	2.81 [1.74–4.52]	**<0.0001**
Pizza	640	1.35 [0.79–2.30]	0.3	1.49 [0.91–2.44]	0.1	3.78 [2.31–6.19]	**<0.0001**	1.38 [0.80–2.39]	0.3
Cakes	644	2.15 [1.28–3.62]	**0.004**	3.47 [2.13–5.66]	**<0.0001**	7.57 [4.57–12.53]	**<0.0001**	4.16 [2.42–7.16]	**<0.0001**
Breakfast Cereals	635	2.11 [1.18–3.79]	**0.01**	2.24 [1.31–3.85]	**0.003**	4.53 [2.65–7.76]	**<0.0001**	2.21 [1.21–4.04]	**0.01**

The reference of the multivariate ordinal logistic regression was the Reference Intakes. The multivariate model was adjusted on sex, age, educational level, level of income, responsibility for grocery shopping, self-estimated diet quality, and self-estimated nutrition knowledge level. HSR: Health Star Rating system; MTL: Multiple Traffic Lights; OR: Odds Ratio; CI: Confidence Interval. Bold values correspond to significant results corrected for multiple testing (*p*-value ≤ 0.05).

**Table 4:** Comparison of the average score and the standard deviation of each statement by FoPL.

Questions	Contributions		Coordinates		Label	v-Test	
	Dimension 1	Dimension 2	Dimension 1	Dimension 2		Dimension 1	Dimension 2
This label is confusing	20.76	9.86	−1.75	0.77	HSR	−0.52	1.30
I like this label	12.49	12.29	1.36	0.86	MTL	−0.35	1.76
This label does not stand out	13.13	14.28	−1.39	0.93	Nutri-Score	3.18	−3.44
This label is easy to understand	17.47	1.66	1.61	0.32			
This label takes too long to understand	18.51	16.97	−1.66	1.02	RIs	−2.26	2.17
This label provides me the information I need	8.46	26.94	1.12	1.28			
I trust this label	9.17	17.99	1.17	1.05	Warning symbol	−0.06	−1.79

## Data Availability

The data used in this study are available on request to the corresponding author.

## References

[1] Global Burden of Disease Study 2019; Seattle, United States: Institute for Health Metrics and Evaluation (IHME) GBD Results Tool|GHDx. http://ghdx.healthdata.org/gbd-results-tool.

[2] Goiana-da-Silva F., Nunes A.M., Miraldo M., Bento A., Breda J., Araújo F.F. (2018). Fiscalidade Ao Serviço Da Saúde Pública: A Experiência Na Tributação Das Bebidas Açucaradas Em Portugal. Acta Médica Port..

[3] Lopes C., Torres D., Oliveira A., Severo M., Alarcão V., Guiomar S., Mota J., Teixeira P., Ramos E., Rodrigues S. (2017). Inquérito Alimentar Nacional e de Atividade Física IAN-AF 2015–2016: Relatório Metodológico.

[4] Direção-Geral da Saúde (2017). Direção de Serviços de Informação e Análise. A Saúde Dos Portugueses 2016.

[5] Grunert K.G., Fernández-Celemín L., Wills J.M., genannt Bonsmann S.S., Nureeva L. (2010). Use and Understanding of Nutrition Information on Food Labels in Six European Countries. J. Public Health.

[6] Gomes S., Nogueira M., Ferreira M., Gregório M.J. (2017). Portuguese Consumers’ Attitudes towards Food Labelling.

[7] Goiana-da-Silva F., Cruz D., Gregório M.J., Nunes A.M., Calhau C., Hercberg S., Rito A., Bento A., Cruz D., Almeida F. (2019). Nutri-Score: A Public Health Tool to Improve Eating Habits in Portugal. Acta Med. Port..

[8] Kanter R., Vanderlee L., Vandevijvere S. (2018). Front-of-Package Nutrition Labelling Policy: Global Progress and Future Directions. Public Health Nutr..

[9] WHO (2004). Global Strategy on Diet, Physical Activity and Health.

[10] Organisation for Economic Co-Operation and Development (2008). Promoting Sustainable Consumption—Good Practices in OECD Countries.

[11] Kleef E.V., Dagevos H. (2015). The Growing Role of Front-of-Pack Nutrition Profile Labeling: A Consumer Perspective on Key Issues and Controversies. Crit. Rev. Food Sci. Nutr..

[12] Vyth E.L., Steenhuis I.H., Roodenburg A.J., Brug J., Seidell J.C. (2010). Front-of-Pack Nutrition Label Stimulates Healthier Product Development: A Quantitative Analysis. Int. J. Behav. Nutr. Phys. Act..

[13] Mhurchu C.N., Eyles H., Choi Y.-H. (2017). Effects of a Voluntary Front-of-Pack Nutrition Labelling System on Packaged Food Reformulation: The Health Star Rating System in New Zealand. Nutrients.

[14] World Health Organization (2017). Tackling NCDs: “Best Buys” and Other Recommended Interventions for the Prevention and Control of Noncommunicable Diseases.

[15] Scrinis G., Parker C. (2016). Front-of-Pack Food Labeling and the Politics of Nutritional Nudges. Law Policy.

[16] Draper A.K., Adamson A.J., Clegg S., Malam S., Rigg M., Duncan S. (2013). Front-of-Pack Nutrition Labelling: Are Multiple Formats a Problem for Consumers?. Eur. J. Public Health.

[17] Europa Summary of EU Legislation (2011). Labeling of Foodstuffs.

[18] Detail|CODEXALIMENTARIUS FAO-WHO. https://www.fao.org/fao-who-codexalimentarius/committees/ewg/detail/en/c/1200590/.

[19] Egnell M., Boutron I., Péneau S., Ducrot P., Touvier M., Galan P., Buscail C., Porcher R., Ravaud P., Hercberg S. (2021). Randomised Controlled Trial in an Experimental Online Supermarket Testing the Effects of Front-of-Pack Nutrition Labelling on Food Purchasing Intentions in a Low-Income Population. BMJ Open.

[20] Egnell M., Ducrot P., Touvier M., Allès B., Hercberg S., Kesse-Guyot E., Julia C. (2018). Objective Understanding of Nutri-Score Front-Of-Package Nutrition Label According to Individual Characteristics of Subjects: Comparisons with Other Format Labels. PLoS ONE.

[21] Cecchini M., Warin L. (2016). Impact of Food Labelling Systems on Food Choices and Eating Behaviours: A Systematic Review and Meta-Analysis of Randomised Studies. Obes. Rev..

[22] Emrich T.E., Qi Y., Lou W.Y., L’Abbe M.R. (2017). Traffic-Light Labels Could Reduce Population Intakes of Calories, Total Fat, Saturated Fat, and Sodium. PLoS ONE.

[23] Deschamps V., Julia C., Salanave B., Verdot C., Hercberg S., Castetbon K. (2015). Score de qualité nutritionnelle des aliments de la Food Standards Agency appliqué aux consommations alimentaires individuelles des adultes en France. Bull. Epidémiologique Hebd..

[24] Julia C., Hercberg S., World Health Organization (2017). Development of a New Front-of-Pack Nutrition Label in France: The Five-Colour Nutri-Score. Public Health Panor..

[25] Ipsos, London Economics Consortium (2013). Consumer Market Study on the Functioning of Voluntary Food Labelling Schemes for Consumers in the European Union EAHC/FWC/2012 86 04.

[26] Egnell M., Talati Z., Galan P., Andreeva V.A., Vandevijvere S., Gombaud M., Dréano-Trécant L., Hercberg S., Pettigrew S., Julia C. (2020). Objective Understanding of the Nutri-Score Front-of-Pack Label by European Consumers and Its Effect on Food Choices: An Online Experimental Study. Int. J. Behav. Nutr. Phys. Act..

[27] Egnell M., Talati Z., Hercberg S., Pettigrew S., Julia C. (2018). Objective Understanding of Front-of-Package Nutrition Labels: An International Comparative Experimental Study across 12 Countries. Nutrients.

[28] Grunert K.G., Wills J.M. (2007). A Review of European Research on Consumer Response to Nutrition Information on Food Labels. J. Public Health.

[29] Morineau A. (1992). Tests et Valeurs-Tests: Application à l’étude de Mastics Utilisés Dans La Fabrication Des Vitraux. Rev. De Stat. Appliquée.

[30] Nielsen A.C. (2012). Battle of the Bulge & Nutrition Labels Healthy Eating Trends Around the World.

[31] Van Kleef E., Van Trijp H., Paeps F., Fernandez-Celemin L. (2008). Consumer Preferences for Front-of-Pack Calories Labelling. Public Health Nutr..

[32] Santos O., Alarcão V., Feteira-Santos R., Fernandes J., Virgolino A., Sena C., Vieira C.P., Gregório M.J., Nogueira P., Graça P. (2020). Impact of Different Front-of-Pack Nutrition Labels on Online Food Choices. Appetite.

[33] Ducrot P., Méjean C., Julia C., Kesse-Guyot E., Touvier M., Fezeu L., Hercberg S., Péneau S. (2015). Effectiveness of Front-of-Pack Nutrition Labels in French Adults: Results from the NutriNet-Sante Cohort Study. PLoS ONE.

[34] Crosetto P., Muller L., Ruffieux B. (2016). Réponses Des Consommateurs à Trois Systèmes d’étiquetage Nutritionnel Face Avant. Cah. De Nutr. Diététique.

[35] Crosetto P., Lacroix A., Muller L., Ruffieux B. (2020). Nutritional and Economic Impact of Five Alternative Front-of-Pack Nutritional Labels: Experimental Evidence. Eur. Rev. Agric. Econ..

[36] Dubois P., Albuquerque P., Allais O., Bonnet C., Bertail P., Combris P., Lahlou S., Rigal N., Ruffieux B., Chandon P. (2021). Effects of Front-of-Pack Labels on the Nutritional Quality of Supermarket Food Purchases: Evidence from a Large-Scale Randomised Controlled Trial. J. Acad. Mark. Sci..

[37] Galan P., Egnell M., Salas-Salvadó J., Babio N., Pettigrew S., Hercberg S., Julia C. (2020). Comprensión de Diferentes Etiquetados Frontales de Los Envases En Población Española: Resultados de Un Estudio Comparativo. Endocrinol. Diabetes Y Nutr..

[38] Fialon M., Egnell M., Talati Z., Galan P., Dréano-Trécant L., Touvier M., Pettigrew S., Hercberg S., Julia C. (2020). Effectiveness of Different Front-of-Pack Nutrition Labels among Italian Consumers: Results from an Online Randomised Controlled Trial. Nutrients.

[39] Gerber M. (2016). Implementing the Mediterranean Diet: A French Perspective and Comparisons with Other Mediterranean Countries. Mediterranean Diet.

[40] Feteira-Santos R., Alarcão V., Santos O., Virgolino A., Fernandes J., Vieira C.P., João Gregório M., Nogueira P., Costa A., Graça P. (2021). Looking Ahead: Health Impact Assessment of Front-of-Pack Nutrition Labelling Schema as a Public Health Measure. Int. J. Environ. Res. Public Health.

